# A Quantum Compass for Materials Discovery: Navigating the Combinatorial Explosion

**DOI:** 10.1021/acscentsci.5c01713

**Published:** 2025-09-20

**Authors:** Kwang S. Kim

**Affiliations:** Department of Chemistry, Ulsan National Institute of Science and Technology, Ulsan 44919, Republic of Korea

## Abstract

A quantum algorithm navigating the immense design space of multivariate porous materials demonstrates a logical and practical roadmap for the future of chemical synthesis.

## Graphical abstract

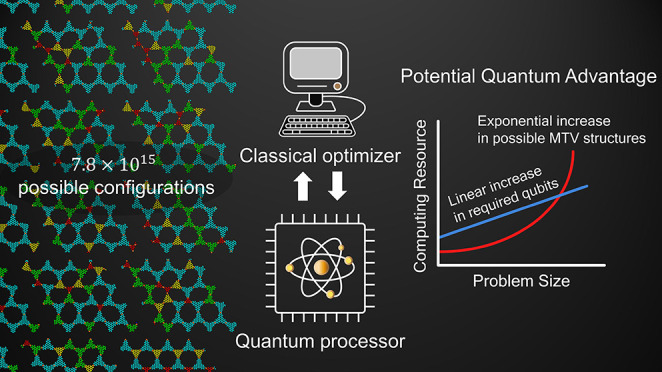

The pursuit of novel materials with specific functionalities has always been a central driver of scientific progress. Yet materials discovery remains one of science’s most formidable challenges, especially for complex systems like multivariate (MTV) porous materials. These sophisticated structures—including metal–organic frameworks (MOFs) and covalent-organic frameworks (COFs)—are built from diverse molecular building blocks. While this compositional variety offers tremendous potential for creating materials with precisely tailored properties, it simultaneously creates a design space so vast that it defies traditional exploration methods.

In this issue of *ACS Central Science*, Jihan Kim and co-workers present what may be the most significant breakthrough in computational materials discovery in decades.

Their paper details the first quantum algorithm specifically engineered to identify optimal chemical configurations for these complex materials, offering both a robust theoretical framework and compelling real-world validation on actual quantum hardware.^1^

Consider the mathematical reality: for a system with just 32 linker sites and eight different linkers, the number of unique structural configurations reaches 7.8 × 10^15^ possibilities. This astronomical figure far exceeds the capacity of even the most powerful classical supercomputers to explore systematically. It is this fundamental “combinatorial explosion” that has long served as an insurmountable bottleneck in rational materials design, forcing researchers to rely on intuition, serendipity, and trial-and-error approaches.

The core innovation lies in reformulating the materials design challenge as a quantum optimization problem. Rather than attempting to brute-force search through impossible numbers of configurations, the authors developed a sophisticated Hamiltonian model that encodes compositional, structural, and balance constraints directly into a quantum system. Instead of tackling the computationally prohibitive many-body Schrödinger equation, they created an ingenious cost function composed of three essential terms. The ratio cost term enforces desired proportions of different linker types, penalizing configurations that deviate from specified compositions. The occupancy cost term ensures physical realism by requiring each linker site to be occupied by exactly one linker. Finally, the balance cost term promotes spatially uniform distribution of linkers by minimizing deviations from stable mean edge lengths.

The key insight driving their computational advantage lies in clever qubit encoding strategies. Different linker types map to quantum states of qubits, allowing the total number of qubits to scale linearly with the product of linker types and sites.

This mapping leverages quantum superposition’s unique ability to simultaneously represent all possible configurations—a scaling advantage that sits at the heart of quantum computing’s revolutionary promise (Figure 1).

**Figure 1:**
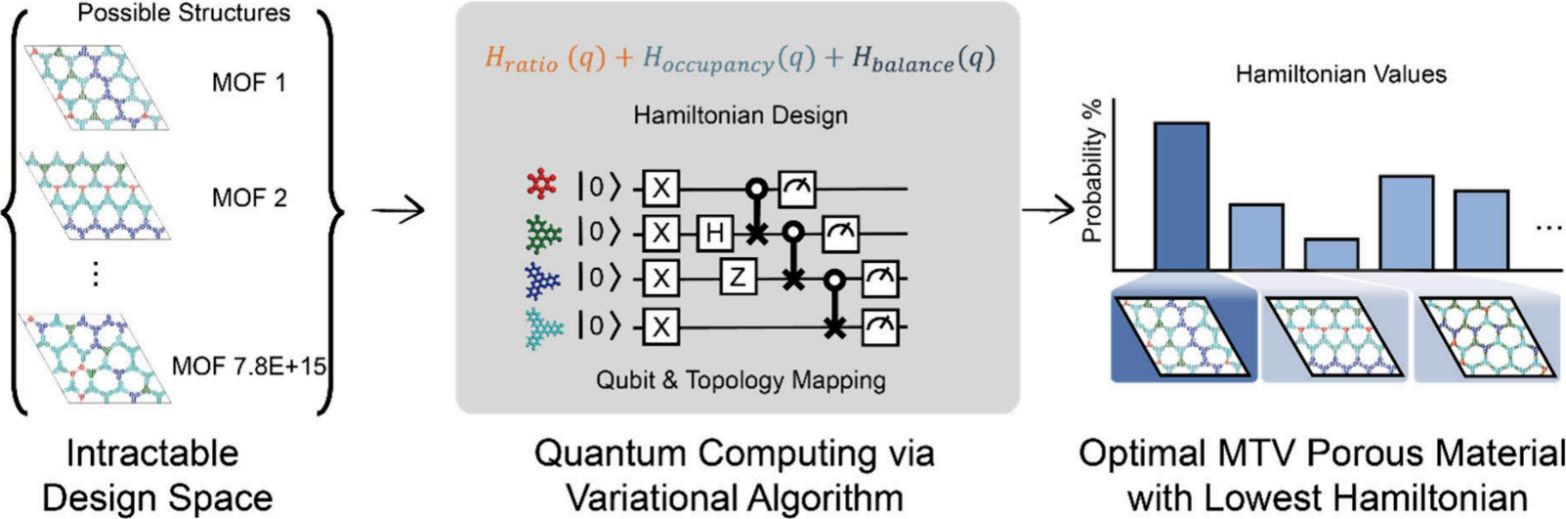
A two-part schematic contrasting the classical problem with the quantum solution. On the left, a chaotic, unoptimized network of different-colored building blocks represents the intractable combinatorial space. On the right, a clean, elegant, and ordered network with the same blocks, overlaid with the image of a quantum circuit, symbolizes the quantum-enabled solution. Adapted from ref 1. Available under a CC-BY 4.0 license. Copyright 2025 Shinyoung Kang, Younghun Kim, and Jihan Kim.

The team employed the variational quantum eigensolver (VQE), a hybrid quantum–classical algorithm well-suited for contemporary quantum hardware.^2^ This approach builds on established variational quantum methodologies that have shown promise across diverse computational challenges.^3^ VQE’s design is crafted for the “noisy intermediate-scale quantum” (NISQ) era, where quantum devices remain prone to noise and errors. The algorithm offloads computationally intensive optimization loops to classical computers while leveraging quantum processors for state preparation and energy measurement. This iterative feedback process guides systems toward ground states that correspond to the most stable material configurations (Figure 2).

**Figure 2:**
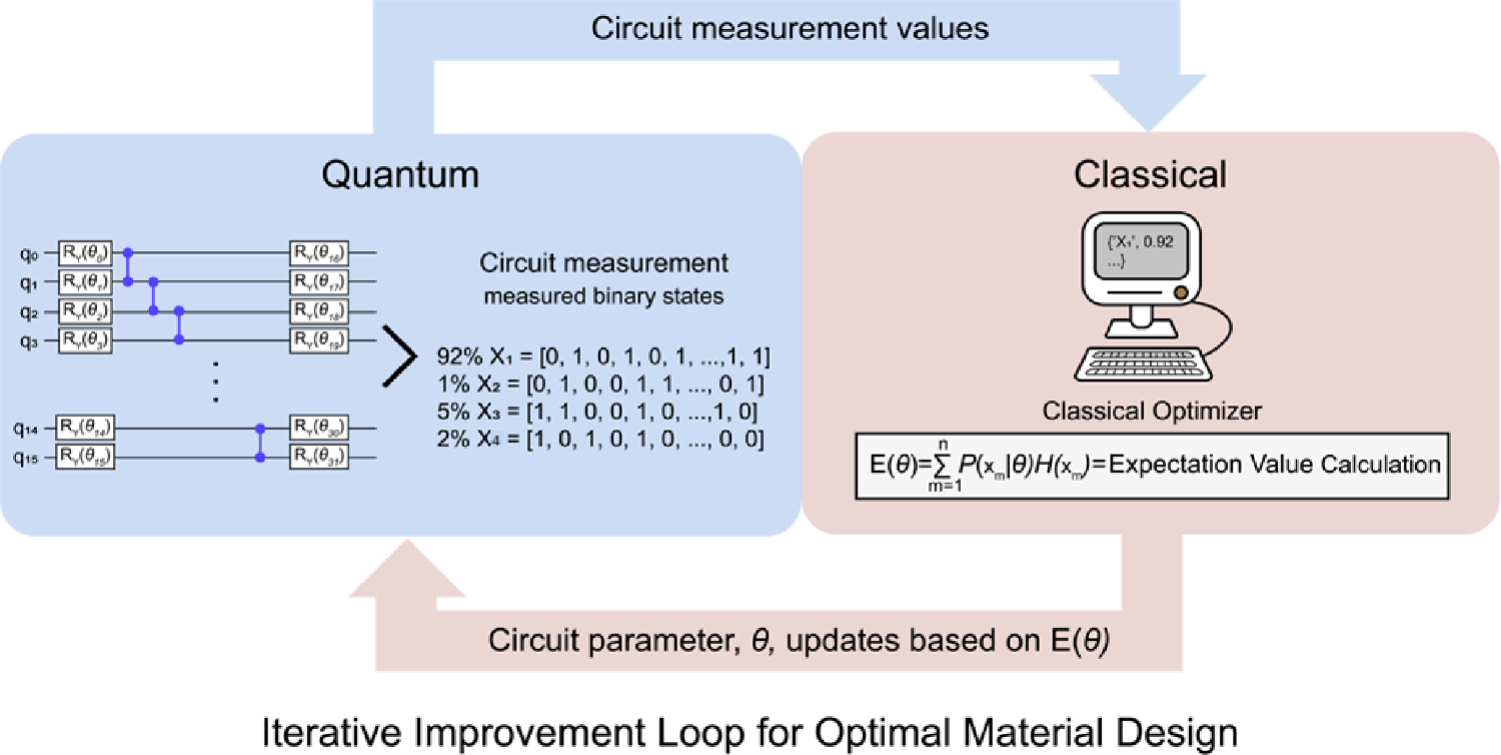
Hybrid VQE workflow. A classical optimizer sends parameters θ to a quantum circuit that prepares and measures material configurations. The quantum measurement results (showing only top four configurations X_1–4_ with their probabilities) are used to calculate energy E­(θ) (the Hamiltonian value) that serves as the material quality score. This energy value feeds back to the classical optimizer, which updates parameters to minimize energy and find the optimal material design through iterative improvement.

What truly distinguishes this research is its rigorous validation, spanning computational simulation to real quantum hardware implementation.

The researchers first demonstrated their algorithm’s reliability by successfully reproducing known ground-state configurations of four diverse experimentally synthesized porous materials with different topologies and linker types. In each case, their algorithm consistently identified the correct experimental structures as the highest-probability outcomes, validating the Hamiltonian’s ability to capture essential geometric and compositional constraints across different material architectures. More remarkably, the team successfully executed VQE calculations on IBM’s ibm_kyiv 127-qubit processor, implementing a 12-qubit system.

The quantum results demonstrated clear convergence trends that closely aligned with classical simulations, proving the algorithm functions effectively even in the presence of real-world noise, gate errors, and decoherence effects. The validation results were impressive across all tested materials, with ground-state probabilities consistently demonstrating successful identification of correct structures.

The authors display refreshing scientific honesty about current limitations. VQE algorithms can become trapped in local minima and require careful optimization strategies. Their coarse-grained approach, while enabling computational scalability, abstracts away atomistic and quantum mechanical details. Consequently, the model cannot capture critical physical properties. However, rather than viewing these as insurmountable obstacles, the authors present a compelling vision for the future. They envision their framework as a scalable “structure generation engine” that can be coupled with classical simulations or machine learning-based property prediction tools.

In this workflow, quantum algorithms would rapidly identify promising material candidates from astronomically large search spaces, serving as an intelligent filter. These quantum-suggested candidates would then be passed to established methods, such as density functional theory calculations and molecular dynamics simulations, for detailed property assessment. The research exemplifies how quantum and classical computing represent complementary rather than competing paradigms. Classical preprocessing proved vital to the quantum algorithm’s success. The authors performed sophisticated analysis of the sensitivity parameter α, which modulates the weight of nontopological connections. They discovered that suboptimal α values could shift ground states and trap quantum algorithms in local minima. By using classical diagnostic tools to identify optimal α values that enhance ground-state energy separation, they ensured reliable convergence to correct solutions.

This pioneering work extends quantum combinatorial optimization principles, previously applied to challenges like protein folding and drug discovery, to porous materials design.

It signals a transformative shift from laborious trial-and-error approaches toward predictive, quantum-assisted design paradigms, opening new avenues for accelerating discovery of materials with tailored properties for applications spanning catalysis, gas separation, energy storage, and beyond.

The implications stretch beyond immediate practical applications. As quantum hardware continues advancing, we can envision quantum computers becoming indispensable tools for chemists and engineers—serving not merely as simulators of known materials but as creative partners in discovering entirely new material classes with unprecedented functionalities.

The work provides a logical roadmap for the scientific community as we venture into this emerging quantum era of rational design. The authors’ frank discussion of current limitations, coupled with their practical vision for hybrid quantum–classical workflows, offers hope that transformative quantum advantages in materials science may be closer than anticipated.

Perhaps most importantly, this research validates that the quantum revolution in chemistry need not await fault-tolerant quantum computers. By thoughtfully combining quantum and classical computational approaches, we can begin realizing meaningful quantum advantages today while building toward an even more powerful quantum-enhanced future for materials discovery.
